# Giant osteomas of the ethmoid and frontal sinuses: Clinical characteristics and review of the literature

**DOI:** 10.3892/ol.2013.1239

**Published:** 2013-03-08

**Authors:** KE-JIA CHENG, SHEN-QING WANG, LIN LIN

**Affiliations:** Department of Otolaryngology, The First Affiliated Hospital, College of Medicine, Zhejiang University, Hangzhou, Zhejiang 310003, P.R. China

**Keywords:** giant osteoma, ethmoid sinus, frontal sinus, clinical characteristics, surgery

## Abstract

Giant osteomas of the ethmoid and frontal sinuses ary very rare, with only a few dozen cases reported in the literature. Given their rarity, the clinical characteristics and treatment of this disease remain controversial. In this study, the clinical presentation and surgical methods used to treat three patients with giant osteomas of the ethmoid and frontal sinuses are described, combined with a review of the literature from 1975 to 2011. In total, 45 patients with giant osteomas arising from the ethmoid and frontal sinuses (including the present cases) have been reported in 41 articles. Headache and ocular signs are the most common symptoms. This disease often leads to intracranial or intraorbital complications. The main treatment for giant osteoma is surgery via an external approach. The outcome of surgery for giant osteoma is good, with rare recurrence, no malignant transformation and few persistent symptoms.

## Introduction

Osteomas occur predominantly in the head and neck region, particularly in the mandible and paranasal sinuses (1). These common benign tumors of the paranasal sinuses affect ∼1% of the population. An osteoma of the paranasal sinuses is usually asymptomatic and found incidentally on imaging examinations. When an osteoma causes symptoms, surgery is required. Osteomas of the paranasal sinuses mainly involve the frontal and ethmoid sinuses. A symptomatic osteoma in the frontal sinus is generally larger than one in the ethmoid sinus because of the greater size of the former. Ethmoid sinus osteomas always show symptoms at an early stage. While osteomas usually range in size from 2 to 30 mm, an osteoma with a diameter >30 mm or weighing >110 g is considered a ‘large’ or ‘giant’ osteoma (2). Giant osteomas of the paranasal sinuses are rare but readily extend into the intraorbital or intracranial cavity, causing serious complications. A giant osteoma of the sinuses usually requires surgical resection. Given its rarity, the clinical characteristics and treatment of this disease remain controversial. In the past 2 years, we treated three patients with giant osteomas of the ethmoid and frontal sinuses. This report describes the clinical presentations and surgical methods used in these three cases and reviews the literature on giant osteomas of the ethmoid and frontal sinuses. The study was approved by the local Ethics Committee of the First Affiliated Hospital of Zhejiang University, Hangzhou, China. Written informed consent was obtained from the patients.

## Case reports

### Case 1

**Figure 1:**
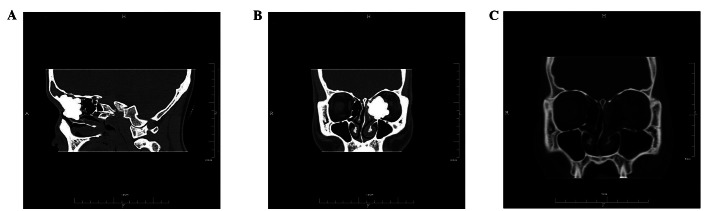
(A) An osseous lesion in the ethmoid sinus extending into the base of the frontal sinus. (B) An osseous lesion in the left ethmoid sinus extending into the orbit. (C) A computed tomography (CT) scan obtained 1 week after surgery revealed no evidence of the osteoma in coronal CT images.

The patient was a 20-year-old male with a 5-year history of hypopsia and ptosis of the left eye. The patient had no associated symptoms, such as epistaxis, rhinorrhea, diplopia or nasal obstruction. He had no history of surgery or trauma and was in good health. An endoscopic examination found no obvious lesion in the nasopharynx or nasal cavity. Computed tomography (CT) revealed a multilobulated 4.0×3.5-cm mass of osseous density occupying the left ethmoid sinus that extended into the left orbit and base of the left frontal sinus. The lesion had displaced the lamina papyracea and compressed the left optic nerve (Fig. 1A and B). The initial diagnosis was a giant osteoma of the ethmoid sinus. The patient’s father had also had an osteoma of the sinuses as a youth and undergone surgery. Proctoscopy was performed to exclude Gardner’s syndrome. The preoperative examination revealed a healthy man with no evidence of lymphadenopathy.

An endoscopic intranasal ethmoidotomy was performed under general anesthesia. Intraoperatively, the lesion was seen to arise from the anterior ethmoid sinus and abutted the lacrimal sac; the margin in the coronal plane extended from the frontal recess to the maxillary ostium. A biopsy of frozen tissue was performed and the histopathological examination confirmed the diagnosis of osteoma. An electric drill was used to resect the tumor in the nasal cavity. However, the anterolateral margin of the osteoma extending into the orbit was not visible. A 3-cm incision was therefore made between the inner canthus and anterior aspect of the nasal bones to expose the anterolateral margin of the tumor. The osteoma was removed using a drill and curette via an external approach combined with intranasal endoscopy. The left periorbital region was intact but was obviously displaced by the osteoma. After removal, the giant osteoma was found to measure ∼4×3×2 cm. A CT performed 1 week postoperatively revealed no evidence of the osteoma (Fig. 1C). Postoperatively, the patient complained of diplopia but this only lasted 3 weeks. The ptosis and visual impairment of the left eye improved. An endoscopic examination showed that the patient was disease-free 13 months postoperatively.

### Case 2

**Figure 2:**
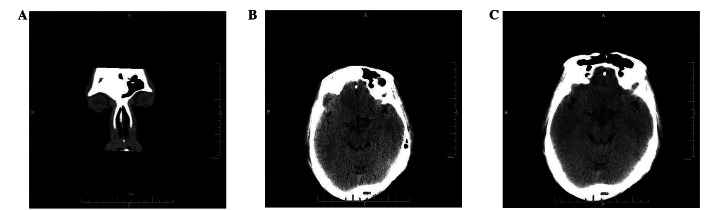
(A) A computed tomography (CT) scan demonstrated a giant bone tumor filling in the right frontal sinus and pushing into the left frontal sinus in coronal CT images. (B) A CT scan demonstrated a giant bone tumor filling the right frontal sinus and pushing into the left frontal sinus in axial CT images. (C) The CT scan revealed total resection of the osteoma 3 months after the surgery in axial CT images.

A 22-year-old male presented with a 3-month history of pain over the region of the nasal bone but no other symptoms. A systemic examination was unremarkable. Endoscopy showed no abnormal findings in the nasal cavity. CT revealed a giant bone tumor filling the right frontal sinus and pushing into the left frontal sinus (Fig. 2A and B).

Surgery was performed under general endotracheal anesthesia. A bicoronal frontal flap was raised with a skin incision along the hairline. Using a drill and bone shears, a frontonasal bone flap was raised. An irregular 6-cm bone-like lesion arose from the roof of the right frontal sinus and pushed against the septum. An intraoperative biopsy of a frozen section showed an osteoma of the frontal sinus. The osteoma was resected completely using a drill and curette. The nasofrontal duct was unblocked. The dura was adherent to the tumor and breached the posterior roof with a cerebrospinal fluid (CSF) leak. Two layers of periosteum and xanthan gum were applied to repair the dura. A bone channel was made in the anterior wall of the frontal sinus, in which the frontal bone flap was inserted to reconstruct the calvarium. Postoperatively, antibiotics were given for 1 week. The patient’s clinical course was uneventful and the nasal root pain was relieved. Three months postoperatively, CT revealed total resection of the osteoma (Fig. 2C). The patient has been disease-free for 16 months and is still being followed up.

### Case 3

**Figure 3:**
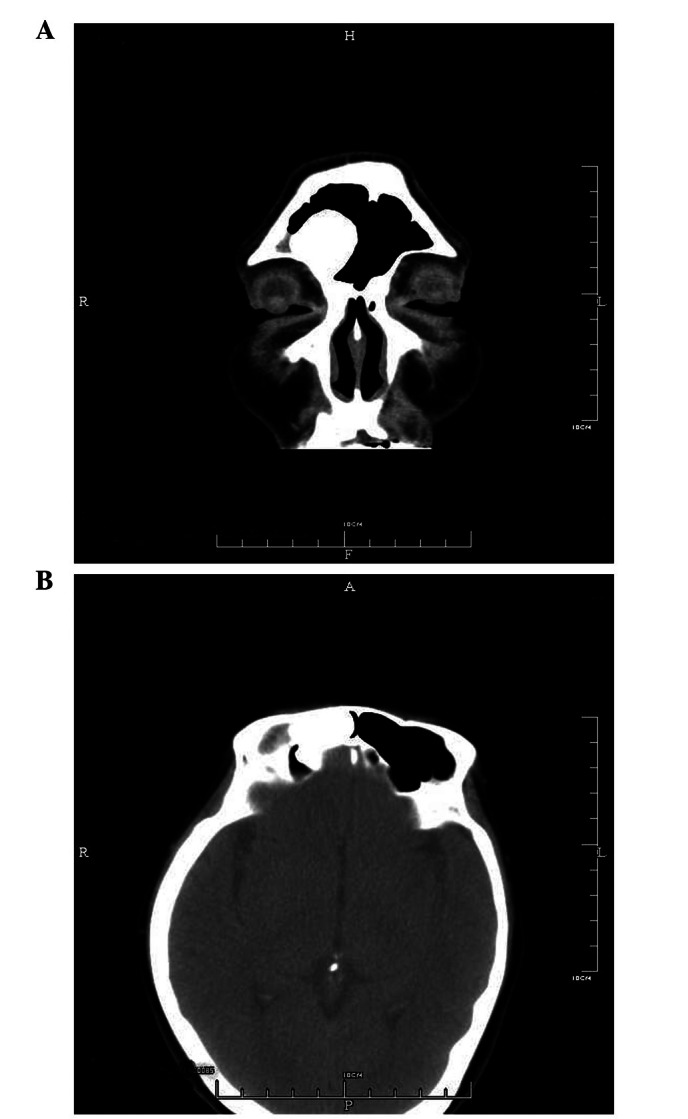
(A) Computed tomography (CT) scans of the sinuses were performed and revealed a 3.5×3-cm extremely dense bony mass with inflammations in the right frontal sinus, approaching the edge of the sinus. (B) An osseous lesion in the right frontal sinus in axial CT images.

A 62-year-old female presented with a 30-day history of a right-side headache. The patient was first examined in the Department of Neurology and was then referred to the Department of Otolaryngology for treatment. The patient had no associated rhinorrhea, epistaxis or nasal obstruction. A systemic examination revealed good health with no evidence of lymphadenopathy or hepatosplenomegaly. The patient had no history of surgery or trauma. An endoscopic examination showed no lesion in the nasal cavity. CT of the sinuses revealed a 3.5×3-cm extremely dense bony mass with inflammation in the right frontal sinus approaching the edge of the sinus (Fig. 3A and B).

Considering the large size and location of the tumor in the CT images, the disease may have resulted in fatal complications. The patient was admitted for excision of the tumor. An endoscopic Draf type IIb frontal sinusotomy was performed. The tumor was removed *en bloc* using a high-speed drill and curette via intranasal endoscopy. The histopathological inspection confirmed the diagnosis of osteoma. The patient was discharged on the fourth postoperative day and has been disease-free for 7 months.

## Discussion

Osteomas are benign tumors often involving the paranasal sinuses, especially the frontal and ethmoid sinuses (3). They usually grow slowly via the continuous formation of bone. Tumors larger than 30 mm in diameter are considered giant tumors (4). Giant osteomas of the paranasal sinuses are rare, with only a few dozen cases reported in the literature. Most of them are located in the frontal or ethmoid sinuses and several are in the maxillary sinuses. To our knowledge, this is the first review of giant osteomas in the frontal and ethmoid sinuses.

The literature on giant osteomas of the ethmoid and frontal sinuses from 1975 to 2011 was reviewed using the keywords ‘osteoma and ethmoid sinus’ or ‘osteoma and frontal sinus’, and 45 patients with giant osteomas in 41 articles were identified (including the cases presented here) (Table I) (5–44). The patients consisted of 28 males and 17 females. The male to female ratio was 1.6:1 and was statistically significant. The patients ranged from 11 to 70 years of age at initial presentation, with a mean age of 39.5 years. In this review, 13 patients (28.9%) were younger than 20 years old, 15 patients (33.3%) were between 20 and 50 years old, 17 patients (37.8%) were between 50 and 70 years old and the disease was not observed in patients over the age of 70. In this review, only four cases (8.9%) involved the sinuses bilaterally, the other 41 cases (91.1%) were unilateral. In the 45 patients, 16 cases (35.6%) were limited to the frontal sinus, 17 cases (37.8%) were limited to the ethmoid sinus and 12 cases (26.7%) involved both the frontal and ethmoid sinuses.

Osteomas are usually asymptomatic and found incidentally on routine radiological examinations. They are diagnosed easily using CT. Osteomas seldom lead to headaches, proptosis, diplopia, dizziness, facial deformity or fatal complications (4). In contrast, giant osteomas more frequently cause symptoms and fatal complications. In this study, headache (48.9%) was the most common symptom, followed by proptosis (40.0%), diplopia (17.8%) and hypopsia (13.3%). This agreed with the report by Erdogan *et al*(4). In patients with giant osteomas of the ethmoid sinuses, ocular signs were much more common (65.5%), and included proptosis, diplopia, hypopsia, epiphora and ptosis. In patients with giant osteomas of the frontal sinuses, headache was often present (60.7%). Complications may occur due to osteoma growth, obstruction of ostia drainage or altered mucus or ciliary function (45). In this review, 42 cases (93.3%) had serious complications, which included 18 cases (40.0%) with involvement of the skull (including intracranial mucoceles in nine cases, pneumocephalus in six cases and cerebral abscesses in four cases) and 24 cases (53.3%) with invasion of the orbit. Obviously, giant osteomas result in complications more often than smaller osteomas. Frontal sinus giant osteomas preponderantly lead to intracranial complications (53.6%), while ethmoid giant osteomas often cause intraorbital complications (68.9%).

The etiology of osteomas is controversial. Traumatic, infective and developmental origins have been considered. Sinusitis or trauma may stimulate osteoblast proliferation within the sinus mucoperiosteum, leading to tumor formation. The developmental theory is based on the fact that many osteomas appear to arise at the junction of the ethmoid and frontal sinus, a location where membranous and cartilaginous tissues meet during embryonic life (23).

In this review, 36 patients (80.0%) had no history of sinusitis or trauma and no obvious etiology. Four patients (8.9%) had a history of surgery, three (6.7%) had a history of head trauma and one (2.2%) had chronic sinusitis. It was postulated that trauma, especially surgical, might be a cause of giant osteomas. In this series, one patient’s father also had an osteoma of the sinuses and underwent surgery as a youth. Given the large number of asymptomatic undiagnosed osteomas, hereditary factors need to be considered in the etiology of this disease. Some authors have reported that osteomas of the paranasal sinuses are usually accompanied by nasal polyps. They inferred that these two diseases might have a similar pathogenesis but only one patient had nasal polyps in the current review. This implies that giant osteomas of the ethmoid and frontal sinuses have no relationship with nasal polyps.

Osteomas may also be a part of Gardner’s syndrome, an autosomal-dominant hereditary disorder characterized by the clinical triad of intestinal polyposis, osteomas and cutaneous and soft tissue tumors (46). In affected individuals, the risk of developing colon cancer approaches 100% (47). Multiple osteomas of the skull may be the initial finding in these patients. When a paranasal sinus osteoma is first diagnosed, Gardner’s syndrome should be excluded with a colonoscopy (48). In this review, none of the patients with giant osteomas had evidence of Gardner’s syndrome.

Grossly, osteomas are round or oval, hard, yellowish-white and well-circumscribed and are attached to the adjacent bone by a broad base or occasionally a small stalk (49). Histologically, osteomas are classified as ivory, mature and mixed types. Ivory osteomas are composed of dense, mature, lamellar bone with little fibrous stroma. Mature osteomas are composed of large trabeculae of mature, lamellar bone with more abundant fibrous stroma and may or may not be surrounded by osteoblasts. Tumors with both ivory and mature features constitute the mixed type (50).

Osteomas of the paranasal sinuses should be differentiated from osteosarcoma, osteoblastoma and fibrous dysplasia using their radiological and pathological features. The most important entity in the differential diagnosis is well-differentiated parosteal osteosarcoma. The neoplastic trabeculae of woven bone in parosteal osteosarcoma are separated by a cellular fibrous stroma that contains occasional mitotic figures. These features are not seen in osteomas (49).

The main treatment for symptomatic osteomas of the sinuses is surgery using an endoscopic or external approach. Considering the slow growth and rarity of recurrence, some authors suggest incomplete resection of the tumor. However, the treatment of asymptomatic cases remains controversial. A conservative approach with regular radiological examinations is usually recommended in asymptomatic cases (23). Surgery is reserved for those asymptomatic lesions involving >50% of the sinus volume, rapidly evolving osteomas (>1 mm/year), intracranial or intraorbital extension, frontal osteomas located in the frontal recess and sphenoid osteomas (51).

Given their size, the giant osteomas in this study were all symptomatic and most caused intracranial or intraorbital complications. The classical method for removing frontal and ethmoid sinus osteomas is still considered to be surgery via an external approach, including bicoronal scalp flap surgery, frontal craniotomy or a lateral rhinotomy (52). In 35 cases (77.8%) in this review series, an external approach alone was used. Recently, endoscopic endonasal resection of paranasal sinus osteomas has become a useful technique in many cases (53). In this review series, a combined approach was chosen in seven cases (15.6%) and osteomas in three cases (6.7%) were resected completely via endoscopic intranasal surgery. With the use of a 70° nasal endoscope and an adequate drill and navigation system, osteomas may be removed safely via an endoscopic approach. Nevertheless, complete resection of giant osteomas via nasal endoscopy alone remains difficult. In this series, only three cases were resected solely via an endoscopic approach: two involving the ethmoid sinus and the other the frontal sinus. Total resection of giant osteomas in the frontal sinus is more difficult than for those in the ethmoid sinus via endoscopy and a Draf type II or III frontal sinusotomy may be required. Giant osteoma surgery often requires reconstruction after removing the tumor. In the current review, 24 cases required reconstruction. However, reconstruction via endoscopy is difficult and an external approach may be required. The outcome of surgery for giant osteoma is good, with only one case of recurrence in this review series. Malignant transformation has not been reported (49). In this review, the majority of symptoms disappeared after surgery, with five patients complaining of transient symptoms for several weeks. In one patient, ptosis persisted postoperatively.

Giant osteomas of the ethmoid and frontal sinuses are rare, with a male predominance and may lead to symptoms and intracranial or intraorbital complications. Headache and ocular signs are the most common symptoms. The main treatment for giant osteoma is surgery via an external approach. Endoscopic *en bloc* resection of these giant osteomas is a new method and is possible depending on the location and size of the osteoma. The outcome of surgery for giant osteoma is good, with rare recurrence, no malignant transformation and few persistent symptoms.

## Figures and Tables

**Table I:** Review of literature of giant osteomas arising from the ethmoid and frontal sinuses (45 patients in 41 articles).

Author/Year (Ref.)	Gender/Age	Etiology	Symptoms	Location	Complications	Treatment	Outcome
Olumide/1975 (5)	M/16	Trauma history	Painful swelling of supraorbital region	LES, LFS	None	ENS	No REC
Wilkes/1979 (6)	M/16	Trauma history	Headache, proptosis, hypopsia, amaurosis	LES	Involving orbit	ENS	No REC
Malcolm/1983 (7)	F/17	Unknown	Proptosis, diplopia	RES	Involving orbit	ENS	No REC
Sternberg/1984 (8)	M/15	Surgical history	Epiphora	LES	Involving orbit	ENS	No REC
Mendelsohn/1984 (9)	F/31	Unknown	Headache	RFS LFS	Pneumocephalus	ENS+RS	No REC
Mugliston/1985 (10)	M/37	Unknown	Headache, swelling of supraorbital region	RFS, LFS, RES, LES	Involving orbit	ENS+RS	No REC
Bushan/1987 (11)	M/25	Unknown	Headache, proptosis, hypopsia	RFS	Involving orbit	ENS	No REC
Schwartz/1990 (12)	M/14	Unknown	Diplopia, proptosis, ptosis	LFS, LES	Involving orbit	ENS+RS	REC at 6 months
Lunardi/1993 (13)	F/20	Unknown	Headache	LFS, LES	Intracranial mucocele	ENS+RS	No REC
Aldren/1993 (14)	F/68	Unknown	Diplopia, proptosis, feeling of pressure	LES, LFS	Involving orbit	ENS	No REC
Shady/1994 (15)	F/17	Unknown	Headache, generalized fatigue	LFS	Cerebral abscess	ENS+RS	No REC
Rappaport/1994 (16)	M/70	Unknown	Headache	RFS	Pneumocephalus	ENS+RS	No REC, 24 h CSF
Brunori/1996 (17)	F/46	Unknown	Headache, blepharospasm	RFS	Intracranial mucocele	ENS+RS	No REC
	M/63	Unknown	Headache, disorientation	LFS	Intracranial mucocele	ENS+RS	No REC
Hehar/1997 (18)	M/59	Unknown	Proptosis, ectropion	RES	Involving orbit	ENS+RS	No REC,
Chang/1997 (19)	F/53	Unknown	Proptosis	RFS	Involving orbit	ENS+RS	4 months facial palsy
Manaka/1998 (20)	M/67	Unknown	Headache	RFS, RES	Intracranial mucocele	ENS	No REC
Nakayama/1998 (21)	F/35	Unknown	Headache	RES	Pneumocephalus	ENS+RS	No REC
Gossios/1999 (22)	F/53	Chronic sinusitis	Proptosis, hypopsia	LFS	Involving orbit	ENS	No REC
Mansour/1999 (23)	F/66	Unknown	Epiphora, proptosis, ptosis, conjunctival hyperemia	RES	Involving orbit, orbital cellulitis	ENS+EIS	No REC
Goldenberg/2000 (24)	M/11	Unknown	Epiphora, proptosis, facial asymmetry	LFS, LES	Involving orbit	ENS	No REC, 2 weeks diplopia
Koyuncu/2000 (25)	M/38	Unknown	Headache	RES, RFS	Cerebral abscess, intracranial mucocele	ENS	No REC
Summers/2001 (26)	M/51	Unknown	Headache, tonic-clonic seizures, ptosis	LFS	Cerebral abscess	ENS+RS	No REC
Bramley/2001 (27)	M/63	Unknown	Diminished short term memory, hemiparesis	RES	Pneumocephalus involving orbit	ENS+RS	No REC
Johnson/2002 (28)	M/62	Unknown	Headache, confusion, drowsiness, nausea	RFS	Pneumocephalus	ENS+RS	No REC
Osma/2003 (29)	M/35	Unknown	Nasal obstruction, epiphora,	RES	Involving orbit	ENS+RS proptosis	No REC
Naraghi/2003 (30)	M/42	Unknown	Proptosis, diplopia	LES	Involving orbit	EIS	No REC
Tsai/2003 (31)	M/20	Surgical history	Diplopia	RES	Involving orbit	ENS	No REC,
Selva/2003 (32)	F/28	Unknown	Proptosis	RES, RFS	Involving orbit	ENS+EIS	3 months ptosis
Nabeshima/2003 (33)	M/54	Unknown	Convulsion	RFS	Intracranial mucocele	ENS+RS	No REC
Akay/2004 (34)	M/20	Unknown	Headache, convulsion	RFS	Intracranial mucocele	ENS+RS	No REC
	M/20	Unknown	Headache, hypopsia	RFS	Intracranial mucocele	ENS+RS	No REC
Karapantzos/2005 (35)	M/59	Unknown	Epiphora, conjunctival hyperemia, proptosis	RES	Involving orbit	ENS	No REC
Panagiotopoulos/2005 (36)	F/36	Surgical history	Headache, proptosis,	LES, LFS	Cerebral abscess	ENS +RS	No REC
			palpebral swelling				
Saetti/2005 (37)	F/28	Unknown	Headache, diplopia, proptosis	RES	Involving orbit	EIS+ RS	No REC
Dispenza/2005 (38)	M/42	Unknown	Headache, swelling of eyelid	LFS, RFS	Palpebral abscess	ENS+ EIS	No REC
Benatiya/2006 (39)	F/18	Unknown	Stony orbital deformity	RES	Involving orbit, chronic dacryocystitis	ENS	No REC
Gerbrandy/2007 (40)	F/39	Unknown	Headache, diplopia, proptosis, exotropia	RES	Involving orbit	ENS+EIS +RS	No REC
Park/2008 (41)	M/68	Unknown	Headache, slowness of speech, confusion	LES	Pneumocephalus	ENS+EIS+RS	No REC
Yiotakis/2008 (42)	M/52	Unknown	Diplopia, proptosis, hypopsia	LES	Nasal polyps involving orbit	ENS+EIS	No REC
Adeleye/2010 (43)	F/32	Surgical history	Tonic-clonic seizures	RFS, RES	Involving orbit	ENS+RS	No REC
Sakamoto/2011 (44)	M/68	Trauma history	Convulsion	LFS, LES	Intracranial mucocele	ENS	No REC
Present study	M/20	Hereditary factor	Hypopsia, ptosis	LES	Involving orbit	ENS+EIS	No REC, 2 weeks diplopia
	M/22	Unknown	Nasal root pain	RFS, LFS	None	ENS+RS	No REC
	F/62	Unknown	Headache	RFS	None	EIS	No REC

ENS, external nasal surgery (including lateral rhinotomy approach and bicoronal scalp flap surgery); EIS, endoscopic intranasal surgery; RS, reconstruction; LES, left ethmoid sinus; RES, right ethmoid sinus, LFS; left frontal sinus; RFS, right frontal sinus; REC, recurrence; CSF, cerebrospinal fluid.

## References

[1] Bulut E, Acikgoz A, Ozan B, Gunhan O (2010). Large peripheral osteoma of the mandible: a case report. Int J Dent.

[2] Izci Y (2005). Management of the large cranial osteoma: experience with 13 adult patients. Acta Neurochir (Wien).

[3] Bignami M, Dallan I, Terranova P, Battaglia P, Miceli S, Castelnuovo P (2007). Frontal sinus osteomas: the window of endonasal endoscopic approach. Rhinology.

[4] Erdogan N, Demir U, Songu M, Ozenler NK, Uluç E, Dirim B (2009). A prospective study of paranasal sinus osteomas in 1889 cases: changing patterns of localization. Laryngoscope.

[5] Olumide AA, Fajemisin AA, Adeloye A (1975). Osteoma of the ethmofrontal sinus. Case report. J Neurosurg.

[6] Wilkes SR, Trautmann JC, DeSanto LW, Campbell RJ (1979). Osteoma an unusual cause of amaurosis fugax. Mayo Clin Proc.

[7] Malcolm WM, Haskell N (1983). Transcoronal removal of an atypical orbitoethmoid osteoma. Plast Reconstr Surg.

[8] Sternberg I, Levine MR (1984). Ethmoidal sinus osteoma - a primary cause of nasolacrimal obstruction and dacryocystorhinostomy failure. Ophthalmic Surg.

[9] Mendelsohn DB, Hertzanu Y, Friedman R (1984). Frontal osteoma with spontaneous subdural and intracerebral pneumatocele. J Laryngol Otol.

[10] Mugliston TA, Stafford N (1985). A cranio-facial approach to large osteomas of the fronto-ethmoidal region. J Laryngol Otol.

[11] Bushan B, Watal G, Ahmed A, Saxena R, Goswami K, Pathania AG (1987). Giant ivory osteoma of frontal sinus. Australas Radiol.

[12] Schwartz MS, Crockett DM (1990). Management of a large frontoethmoid osteoma with sinus cranialization and cranial bone graft reconstruction. Int J Pediatr Otorhinolaryngol.

[13] Lunardi P, Missori P, Di Lorenzo N, Fortuna A (1993). Giant intracranial mucocele secondary to osteoma of the frontal sinuses: report of two cases and review of the literature. Surg Neurol.

[14] Aldren CP, Soames JV, Birchall JP (1993). Bony remodelling in an osteoma of the paranasal sinuses. J Laryngol Otol.

[15] Shady JA, Bland LI, Kazee AM, Pilcher WH (1994). Osteoma of the frontoethmoidal sinus with secondary brain abscess and intracranial mucocele: case report. Neurosurgery.

[16] Rappaport JM, Attia EL (1994). Pneumocephalus in frontal sinus osteoma: a case report. J Otolaryngol.

[17] Brunori A, de Santis S, Bruni P, Delitala A, Giuffre R, Chiappetta F (1996). Life threatening intracranial complications of frontal sinus osteoma: report of two cases. Acta Neurochir (Wien).

[18] Hehar SS, Jones NS (1997). Fronto-ethmoid osteoma: the place of surgery. J Laryngol Otol.

[19] Chang SC, Chen PK, Chen YR, Chang CN (1997). Treatment of frontal sinus osteoma using a craniofacial approach. Ann Plast Surg.

[20] Manaka H, Tokoro K, Sakata K, Ono A, Yamamoto I (1998). Intradural extension of mucocele complicating frontoethmoid sinus osteoma: case report. Surg Neurol.

[21] Nakayama Y, Tanaka A, Ueno Y, Naritomi K, Yoshinaga S (1998). Pneumocephalus associated with ethmoidal sinus osteoma. Neurol Med Chir (Tokyo).

[22] Gossios K, Bai M, Psilas K (1999). Giant aggressive osteoma of the frontal sinus. Clin Radiol.

[23] Mansour AM, Salti H, Uwaydat S, Dakroub R, Bashshour Z (1999). Ethmoid sinus osteoma presenting as epiphora and orbital cellulitis: case report and literature review. Surv Ophthalmol.

[24] Goldenberg D, Gilboa M, Danino J, Flax-Goldenberg R, Miller B, Joachims HZ (2000). A large ethmoido-orbital osteoma presenting with epiphora in an 11-year-old boy. J Pediatr Ophthalmol Strabismus.

[25] Koyuncu M, Belet U, Seşen T, Tanyeri Y, Simşek M (2000). Huge osteoma of the frontoethmoidal sinus with secondary brain abscess. Auris Nasus Larynx.

[26] Summers LE, Mascott CR, Tompkins JR, Richardson DE (2001). Frontal sinus osteoma associated with cerebral abscess formation: a case report. Surg Neurol.

[27] Bramley DC, Ghosh S (2001). Tension pneumocephalus attributable to an ethmoid osteoma presenting as a stroke in evolution: an unusual presentation. Emerg Med J.

[28] Johnson D, Tan L (2002). Intraparenchymal tension pneumatocele complicating frontal sinus osteoma: case report. Neurosurgery.

[29] Osma U, Yaldiz M, Tekin M, Topcu I (2003). Giant ethmoid osteoma with orbital extension presenting with epiphora. Rhinology.

[30] Naraghi M, Kashfi A (2003). Endonasal endoscopic resection of ethmoido-orbital osteoma compressing the optic nerve. Am J Otolaryngol.

[31] Tsai CJ, Ho CY, Lin CZ (2003). A huge osteoma of paranasal sinuses with intraorbital extension presenting as diplopia. J Chin Med Assoc.

[32] Selva D, Chen C, Wormald PJ (2003). Frontoethmoidal osteoma: a stereotactic-assisted sino-orbital approach. Ophthal Plast Reconstr Surg.

[33] Nabeshima K, Marutsuka K, Shimao Y, Uehara H, Kodama T (2003). Osteoma of the frontal sinus complicated by intracranial mucocele. Pathol Int.

[34] Akay KM, Ongürü O, Sirin S, Celasun B, Gönül E, Timurkaynak E (2004). Association of paranasal sinus osteoma and intracranial mucocele. Neurol Med Chir (Tokyo).

[35] Karapantzos I, Detorakis ET, Drakonaki EE, Ganasouli DL, Danielides V, Kozobolis VP (2005). Ethmoidal osteoma with intra-orbital extension: excision through a transcutaneous paranasal incision. Acta Ophthaloml Scand.

[36] Panagiotopoulos V, Tzortzidis F, Partheni M, Iliadis H, Fratzoglou M (2005). Giant osteoma of the frontoethmoidal sinus associated with two cerebral abscesses. Br J Oral Maxillofac Surg.

[37] Saetti R, Silvestrini M, Narne S (2005). Ethmoid osteoma with frontal and orbital extension: endoscopic removal and resconstruction. Acta Otolaryngol.

[38] Dispenza C, Saraniti C, Ferrara S, Martines F, Caramanna C, Salzano FA (2005). Frontal sinus osteoma and palpebral abscess: case report. Rev Laryngol Otol Rhinol (Bord).

[39] Benatiya Andaloussi I, Touiza E, Bhallil S, Oudidi A, Bouayed MA, Daoudi K (2006). Orbital osteoma: three case reports. Bull Soc Belge Ophtalmol.

[40] Gerbrandy SJ, Saeed P, Fokkens WJ (2007). Endoscopic amd transfornix removal of a giant orbital-ethmoidal osteoma. Orbit.

[41] Park MC, Goldman MA, Donahue JE, Tung GA, Goel R, Sampath P (2008). Endonasal ethmoidectomy and bifrontal craniotomy with craniofacial approach for resection of frontoethmoidal osteoma causing tension pneumocephalus. Skull Base.

[42] Yiotakis I, Eleftheriadou A, Giotakis E, Manolopoulos L, Ferekidou E, Kandiloros D (2008). Resection of giant ethmoid osteoma with orbital and skull base extension followed by duraplasty. World J Surg Oncol.

[43] Adeleye AO (2010). A giant complex fronto-ethmoidal ivory osteoma: surgical technique in a resource-limited practice. Surg Neurol Int.

[44] Sakamoto H, Tanaka T, Kato N, Arai T, Hasegawa Y, Abe T (2011). Frontal sinus mucocele with intracranial extension associated with osteoma in the anterior cranial fossa. Neurol Med Chir (Tokyo).

[45] Gil-Carcedo LM, Gil-Carcedo ES, Vallejo LA, de Campos JM, Herrero D (2011). Frontal osteomas: standardising therapeutic indications. J Laryngol Otol.

[46] Alexander AA, Patel AA, Odland R (2007). Paranasal sinus osteomas and Gardner’s syndrome. Ann Otol Rhinol Laryngol.

[47] Daneshi A, Jalessi M, Heshmatzade-Behzadi A (2010). Middle turbinate osteoma. Clin Exp Otorhinolaryngol.

[48] Lee BD, Lee W, Oh SH, Min SK, Kim EC (2009). A case report of Gardner syndrome with hereditary widespread osteomatous jaw lesions. Oral Surg Oral Med Oral Pathol Oral Radiol Endod.

[49] Nielsen GP, Rosenberg AE (2007). Update on bone forming tumors of the head and neck. Head Neck Pathol.

[50] McHugh JB, Mukherji SK, Lucas DR (2009). Sino-orbital osteoma:a clinicopathologic study of 45 surgically treated cases with emphasis on tumors with osteoblastoma-like features. Arch Pathol Lab Med.

[51] Castelnuovo P, Valentini V, Giovannetti F, Bignami M, Cassoni A, Iannetti G (2008). Osteomas of the maxillofacial district: endoscopic surgery versus open surgery. J Craniofac Surg.

[52] Ledderose GJ, Betz CS, Stelter K, Leunig A (2011). Surgical management of osteomas of the frontal recess and sinus: extending the limits of the endoscopic approach. Eur Arch Otorhinolaryngol.

[53] Lund VJ, Stammberger H, Nicolai P (2010). European position paper on endoscopic management of tumours of the nose, paranasal sinuses and skull base. Rhinol Suppl.

